# ADVANCED OLD AGE: NARRATIVES, NEEDS, AND NOTIONS

**DOI:** 10.1093/geroni/igae098.1136

**Published:** 2024-12-31

**Authors:** Elaine Eliopoulos, Chris Gilleard

**Affiliations:** Private Counsel-Committee for Public Counsel Services (contract), Weston, Massachusetts, United States; University College London, London, England, United Kingdom

## Abstract

Longevity trends worldwide have highlighted the need to advance theoretical development of advanced old age beyond a singularly focused decline narrative. This narrative contributes to a doomed sense of old age without consideration for the subjectivities which may contribute to a deeper understanding of the experience of advanced old age beyond its bodily capabilities. This paper reports findings from a qualitative study depicting personal narratives elicited from 23 individuals aged 80 to 102 living on the San Juan Islands in the state of Washington. Using a mix of photo-elicitation and semi-structured interviews, participants narratives reflected a specific focus on their needs and interests despite a range of corporeal challenges. Those needs and interests foregrounded a sense of community experienced in diverse ways on their island. The participants’ focus on community and belonging gives rise to the benefits of embracing new models of advanced old age which capture a sense of contented engagement with both nature and community that contrasts with the dominant discourse of decline and offers new notions of advanced old age. These new notions may enhance both quality of life and directly inform policy designed to address quality.

